# Attention-aware Deep Learning Models for Dermoscopic Image Classification for Skin Disease Diagnosis

**DOI:** 10.2174/0115734056332443241129113146

**Published:** 2025-01-02

**Authors:** Malliga Subramanian, Kogilavani Shanmugavadivel, Sudha Thangaraj, Jaehyuk Cho, Sathishkumar VE

**Affiliations:** 1 Department of Computer Science and Engineering, Kongu Engineering College, Perundurai, Erode, Tamil Nadu, India; 2 Department of Artificial Intelligence, Kongu Engineering College, Perundurai, Erode, Tamil Nadu, India; 3 Department of Software Engineering & Division of Electronics and Information Engineering, Jeonbuk National University, Jeonju-si, 54896, Republic of Korea; 4 School of Engineering and Technology, Sunway University, No. 5, Jalan Universiti, Bandar Sunway, 47500 Selangor Darul Ehsan, Kuala Lumpur, Malaysia

**Keywords:** Skin lesion, CNN, EfficientNetB3, VGG19, ResNet-152, RegNetX, Attention mechanism, Bayesian optimization

## Abstract

**Background:**

The skin, being the largest organ in the human body, plays a vital protective role. Skin lesions are changes in the appearance of the skin, such as bumps, sores, lumps, patches, and discoloration. If not identified and treated promptly, skin lesion diseases would become a serious and worrisome problem for society due to their detrimental effects. However, visually inspecting skin lesions during medical examinations can be challenging due to their similarities.

**Objective:**

The proposed research aimed at leveraging technological advancements, particularly deep learning methods, to analyze dermoscopic images of skin lesions and make accurate predictions, thereby aiding in diagnosis.

**Methods:**

The proposed study utilized four pre-trained CNN architectures, RegNetX, EfficientNetB3, VGG19, and ResNet-152, for the multi-class classification of seven types of skin diseases based on dermoscopic images. The significant finding of this study was the integration of attention mechanisms, specifically channel-wise and spatial attention, into these CNN variants. These mechanisms allowed the models to focus on the most relevant regions of the dermoscopic images, enhancing feature extraction and improving classification accuracy. Hyperparameters of the models were optimized using Bayesian optimization, a probabilistic model-based technique that efficiently uses the hyperparameter space to find the optimal configuration for the developed models.

**Results:**

The performance of these models was evaluated, and it was found that RegNetX outperformed the other models with an accuracy of 98.61%. RegNetX showed robust performance when integrated with both channel-wise and spatial attention mechanisms, indicating its effectiveness in focusing on significant features within the dermoscopic images.

**Conclusion:**

The results demonstrated the effectiveness of attention-aware deep learning models in accurately classifying various skin diseases from dermoscopic images. By integrating attention mechanisms, these models can focus on the most relevant features within the images, thereby improving their classification accuracy. The results confirmed that RegNetX, integrated with optimized attention mechanisms, can provide robust, accurate diagnoses, which is critical for early detection and treatment of skin diseases.

## 1 INTRODUCTION

Skin diseases represent a major public health challenge globally, affecting millions of people across various demographics and regions. The prevalence of skin diseases, ranging from benign conditions, such as moles and cysts, to malignant tumors has been steadily increasing. Malignant tumors, which include skin cancers like melanoma, squamous cell carcinoma, basal cell carcinoma, and others, are harmful diseases [1]. Skin lesion classification involves categorizing lesions into different types, such as melanoma, basal cell carcinoma, squamous cell carcinoma, nevus, *etc*. However, although basal cell carcinoma is not usually fatal, it is the most prevalent type of skin cancer and has a significant impact on healthcare resources. Undiagnosed skin diseases can have a detrimental psycho-social impact and lower quality of life for those who experience such diseases [2, 3]. Hence, early and accurate diagnosis of skin lesions is crucial for timely intervention and effective treatment. Yet, it remains a challenging task due to the array of dermatological conditions and the complexity of visual cues present in dermoscopic images.

Dermoscopy, a non-invasive imaging technique that provides magnified visualization of skin structures, has emerged as a widely accepted standard for skin lesion examination. Despite its advantages over traditional visual inspection, the interpretation of dermoscopic images remains subjective and highly dependent on the expertise of the clinicians. This necessitates the need for automated image-analyzing techniques to support clinical decision-making. With the advancement of Artificial Intelligence (AI), particularly machine and deep learning algorithms, the accurate detection and classification of skin diseases from dermoscopic images have become increasingly achievable [4, 5]. The features extracted from dermoscopic images, such as texture, color, shape, and spatial arrangements, can be used as input to AI models for classification. These models learn to distinguish between different types of lesions based on the learned features, aiding in accurate diagnosis and treatment [6]. By leveraging machine and deep learning techniques, dermatologists can enhance their diagnostic capabilities, thus potentially reducing healthcare costs.

Deep learning models, particularly Convolutional Neural Networks (CNNs), have demonstrated remarkable success in medical image analysis due to their ability to automatically learn hierarchical feature representations directly from raw input data. These models can capture complex patterns and textures that are indicative of different skin diseases, which traditional machine-learning models with handcrafted features often fail to detect [6, 7]. Despite these advancements, the inherent variability and complexity of skin lesions pose significant challenges to CNNs, necessitating the development of more sophisticated models that can enhance feature extraction and improve classification accuracy.

In recent years, the emergence of deep learning techniques, such as CNNs, has revolutionized the field of medical image analysis, offering promising avenues for automated skin disease diagnosis [7, 8]. In this context, attention-aware deep learning models have emerged as a promising approach for dermoscopic image classification in skin disease diagnosis [9, 10]. Attention mechanisms, inspired by human visual attention, allow models to selectively focus on the most relevant regions of an image, thereby enhancing their ability to distinguish between similar patterns and features. These mechanisms enable the model to weigh the importance of different spatial regions or channels within an image, ensuring that significant features are effectively highlighted during the learning process. By incorporating attention mechanisms into CNN architectures, these models can selectively focus on specific areas of interest within the images. This attention-driven approach not only improves classification accuracy but also helps dermatologists understand the rationale behind the classification results.

This study aims to advance the field of automated skin disease diagnosis by exploring the integration of attention mechanisms into CNN-based models for dermoscopic image classification. We specifically focus on the application of both spatial and channel-wise attention mechanisms to enhance the feature extraction capabilities of CNNs, thereby improving their diagnostic performance. Through rigorous experimentation on diverse dermatological datasets, this research evaluates the efficacy of various attention-aware deep-learning models in accurately identifying and classifying multiple types of skin lesions. The findings of this study are expected to provide valuable insights into the development of more effective AI-based diagnostic tools, potentially transforming clinical practice and reducing the burden on healthcare systems.

### 1.1 Novelty of the Proposed Work

The novelty lies in the strategic integration of both spatial and channel-wise attention mechanisms within pre-trained CNN models. Unlike traditional approaches, which often rely on standard CNN architectures without attention, this research emphasizes selectively enhancing the focus on critical regions and features within dermoscopic images.

#### 1.1.1 Spatial Attention Mechanisms

These mechanisms are designed to prioritize regions within an image, enabling the models to focus on localized patterns and anomalies that are indicative of specific skin conditions. This is particularly important for skin disease diagnosis, where localized image features, such as irregular borders, are crucial for distinguishing between benign and malignant lesions.

#### 1.1.2 Channel-wise Attention Mechanisms

These mechanisms allow the model to learn the importance of different feature channels, which correspond to various learned filters within the network. By weighting these channels appropriately, the model becomes more sensitive to complex feature interactions and can better capture the skin lesion patterns.

Furthermore, the research presents an approach to transfer learning by selectively unfreezing and fine-tuning the top layers of pre-trained CNN models. This technique ensures that the higher-level feature representations, which are important for capturing domain-specific details, are effectively adapted to the task of skin lesion classification. This selective fine-tuning approach enhances the ability of the models to generalize from pre-trained knowledge to the new task of skin disease classification.

Additionally, we employ Bayesian optimization for hyperparameter tuning, which provides a more systematic search process compared to grid or random search methods. This helps the models perform best, providing strong diagnostic capabilities for different types of skin diseases.

### 1.2 Main Contributions of the Proposed Work

The key contributions of this work are as follows:

#### 1.2.1 Development of Classifiers

The research leverages both traditional CNN architectures and state-of-the-art deep learning models integrated with attention mechanisms. These models are specifically tailored for the multi-class classification of skin lesions, addressing a broader range of dermatological conditions.

#### 1.2.2 Transfer Learning Approach

In this approach, fine-tuning top layers in pre-trained models for higher-order feature representations enhance their relevance to specific dermatological datasets. This approach improves the adaptability and diagnostic accuracy of the classifiers, especially in handling complex and varied skin lesion images.

#### 1.2.3 Incorporation of Dual Attention Mechanisms

By integrating both spatial and channel-wise attention mechanisms, the research significantly improves the feature extraction and focusing capabilities, leading to better performance in distinguishing between different types of skin lesions.

#### 1.2.4 Optimization through Bayesian Search

The fine-tuning of hyperparameters *via* Bayesian optimization ensures that the attention-enhanced models are not only accurate but also computationally efficient, maximizing their applicability.

In the subsequent sections of this paper, we provide a comprehensive overview of related work in skin disease diagnosis and deep learning-based image classification in Section

2. Subsequently, in Section 3, we discuss the dataset selection, model training, and transfer learning and then present the implementation of attention-aware deep learning models for dermoscopic image classification. Then, we present the results of our experiments and provide insights into the performance and effectiveness of attention-aware deep learning models for skin disease diagnosis in Section 4. Finally, we conclude our work and provide the scope for future work in Section 5.

### 2 LITERATURE SURVEY

Skin disease detection has received attention in recent years owing to its importance in early diagnosis and treatment. The advent of CNN has revolutionized the field of medical image analysis [11-14]. With the proliferation of digital imaging technologies, CNN-based approaches have emerged as powerful tools for automating the detection and classification of various skin diseases. This section presents a brief overview of recent works on skin disease detection. We have reorganized the literature review to cover machine learning models first, followed by deep learning models. Then, transformer models, which have gained prominence recently, and studies utilizing attention-based mechanisms are explored.

In a review [15], the authors presented an overview of the state-of-the-art techniques and provided a comparative analysis concerning a number of the core image processing operations, including image acquisition, pre-processing, and segmentation. The methods that have been suggested to accomplish these objectives were identified and examined in this work. Rasool *et al*. [16] provided an overview of skin diseases, their types, available datasets, and data preprocessing techniques and delved into the paradigm of deep learning, highlighting popular methods used in skin disease diagnosis research. The primary objective of this study was to conduct a systematic literature review of skin disease detection using deep learning methodologies employed in recent research. The findings suggest that deep learning approaches consistently yield more accurate results compared to dermatologists, various machine-based therapy strategies, and other classification methods in the domain of skin disease image recognition. Li *et al*. [17] presented a review of deep learning methods and their utilization in skin disease diagnosis. It provides an overview of skin diseases and image acquisition techniques in dermatology and discusses several publicly accessible skin datasets. Following this, it introduces the concept of deep learning and reviews prominent architectures and popular frameworks for implementing deep learning algorithms. This work concludes with a discussion on performance evaluation metrics. The use of various machine learning algorithms, including supervised, unsupervised, and deep learning models, for analyzing skin images was presented in a study [18]. A review of novel approaches that incorporate advanced techniques, such as transfer learning, data augmentation, and ensemble methods, to enhance model performance was presented. The potential future directions for AI in skin cancer detection, including the integration of multimodal data (combining dermoscopic images with patient history and genetic information) to improve diagnostic accuracy, were explored [19]. This work highlighted the potential for AI-based tools to assist dermatologists in clinical settings by providing a second opinion or serving as a triage tool to prioritize high-risk cases.

Nasir *et al*. [20] proposed approaches that integrate preprocessing, lesion segmentation, feature extraction, feature selection, and classification. A hybrid technique has been employed for lesion segmentation, and the additive law of probability has been applied to fuse the results. Following the use of the Boltzmann entropy approach, the fused features were chosen, and the chosen features were then categorized using SVM. The publicly available data set PH2 was used to examine this strategy, and the findings showed a sensitivity of 97.7%, specificity of 96.7%, accuracy of 97.5%, and F1-score of 97.5%. Bordoloi *et al*. [21] presented an evolutionary model for skin disease classification and detection, utilizing machine learning and image processing techniques. The model employed Support Vector Machine (SVM), K-Nearest Neighbors (KNN), and Random Forest (RF) algorithms for image categorization and detection. The results indicated that the proposed methodology is effective in accurately identifying skin diseases through image analysis. Specifically, the SVM algorithm achieved an accuracy of 98.8%, while the KNN algorithm demonstrated a sensitivity of 91% and a specificity of 99%.

Bhardwaj *et al*. [22] discussed the difficulty of accurately and quickly diagnosing skin cancer and suggested a neural network model based on SVM to classify skin diseases. On the ISIC 2019 dataset, their model produced an accuracy of 86%. Aldera and Othman [23] introduced a model to diagnose acne, cherry angioma, melanoma, and psoriasis from images of affected skin. This model comprises five steps: image acquisition, preprocessing, segmentation, feature extraction, and classification. The evaluation of the model involves employing machine learning algorithms, namely SVM, RF, and KNN, achieving an accuracy of 90.7%, 84.2%, and 67.1%, respectively. Ahammed *et al*. [24] introduced a digital hair removal technique for skin lesion analysis, integrating morphological filtering, inpainting, and Gaussian filtering to enhance image quality. It employs automatic GrabCut segmentation to isolate lesions and utilizes the Gray Level Co-occurrence Matrix (GLCM) and statistical features for pattern extraction. Three machine learning classifiers, Decision Tree, SVM, and KNN, are applied to classify skin images into various categories, and the validation is conducted using two standard datasets, ISIC 2019 and HAM10000, with SVM demonstrating slightly superior performance compared to other classifiers. A novel automated framework is introduced for multiclass skin lesion classification, involving a series of steps, including augmentation, fine-tuning, and transfer learning [25]. Subsequently, feature extraction is conducted, followed by fusion using a modified serial-based approach. The fused vector is then refined by employing a skewness-controlled SVR approach to select the best features. The final selected features are classified using various machine learning algorithms, with the selection based on accuracy values. Experimental validation is performed using the augmented HAM10000 dataset, resulting in an accuracy of 91.7%.

Inthiyaz *et al*. [6] recommended an automated approach based on CNN for diagnosing and categorizing skin conditions using machine learning classification. Their method employed computational techniques to analyze, process, and characterize images by considering various image features. The study extracted significant features from skin images and classified them, achieving an accuracy of 87%. Taouil and Romdhane *et al*. [26] employed three distinct methods for automatic segmentation: thresholding, morphology functions, and active contours. They quantified malignancy signs using parameters that encapsulate geometric and photometric characteristics of the lesions. The most discriminative parameters were selected for classification. This study evaluated its approach using various instances of color skin lesion images obtained from a digitized database curated by expert dermatologists. Shanthi *et al*. [27] introduced a computer vision-based method for detecting four types of skin diseases and employed CNNs with a focus on skin disease detection, utilizing around 11 layers, including Convolution, Activation, Pooling, Fully Connected, and Soft-Max Classifier. The models were tested using images from the DermNet database, encompassing various skin diseases, with a focus on four specific types. The proposed CNN Classifier achieved an accuracy ranging from 98.6% to 99.04%.

Leyva *et al*. [28] proposed a novel approach for diagnosing ten types of skin lesions using Fourier spectral information derived from images in an additive color model. The spectral data, along with correlation coefficients between different lesion classes, are utilized as input signals for an artificial neural network. The proposed model achieved high classification performance with an accuracy of 99.33%, precision of 94.16%, sensitivity of 92.9%, and specificity of 99.63%. A CNN was trained from images in a study by Esteva *et al*. [29] utilizing pixels and disease labels as inputs. A dataset with 129,450 clinical images encompassing 2,032 distinct diseases was used to train the CNN. Using biopsy-proven clinical images, the performance was evaluated against 21 board-certified dermatologists in two binary classification tasks: separating malignant melanomas from benign nevi and keratinocyte carcinomas from benign seborrheic keratoses. In both tests, CNN performed better than all tested experts, proving that it could classify skin cancer with a degree of proficiency similar to a dermatologist. A novel framework is developed by fine-tuning layers of ResNet152 and InceptionResNet-V2 using a triplet loss function [30]. This framework learns embeddings from input images, mapping them into Euclidean space using deep ResNet152 and InceptionResNet-V2. Subsequently, the L-2 distance between corresponding images in Euclidean space is computed to extract discriminative features of skin disease images through the triplet loss function. Finally, the classification of input images is performed based on these L-2 distances. Experimental results and analysis demonstrate the effectiveness of the proposed framework, achieving superior accuracy compared to many existing approaches in skin disease tasks.

Antari *et al*. [31] developed a deep CNN model featuring multiple layers and various filter sizes, with a reduction in filters and parameters to enhance efficiency. For testing, dermoscopic images from the International Skin Imaging Collaboration databases (ISIC-17, ISIC-18, and ISIC-19) were used. The precision, sensitivity, and specificity of the experimental findings were determined. It is noteworthy that in ISIC-17, it scores 94% accuracy, 93% sensitivity, and 91% specificity. In a study [32], various experiments were conducted with a range of neural networks, including PNASNet-5-Large, InceptionResNetV2, SENet154, and InceptionV4. Dermoscopic images were fed into the network after being pre-processed. After testing on the ISIC-2018 dataset, the models produced a validation score of 0.76 for the PNASNet-5-Large model. Singh *et al*. [4] developed a system combining metaheuristic optimization techniques with AI-based classifiers to improve skin disease detection and diagnosis. By extracting features from pre-processed images and employing various learning models, they found that hyperparameter tuning significantly enhanced model accuracy, with the deep neural network achieving the highest accuracy of 90.56% under Bayesian search cross-validation.

Cai *et al*. [2] introduced a Vision Transformer (ViT) model to extract deep features from images and designed a Soft Label Encoder (SLE) for embedding image metadata. This work proposed a Mutual Attention block in the decoder part to effectively fuse image and metadata features. The model was evaluated on skin disease and ISIC 2018 benchmark datasets, achieving an accuracy of 93.81%. Vachmanus *et al*. [33] introduced DeepMetaForge, a deep-learning framework for skin cancer detection from images with metadata. The framework utilized a pre-trained vision transformer for image encoding. The experimental results on multiple public datasets demonstrated that the optimal configuration of DeepMetaForge achieved a macro-average F1 score of 87.1%. Desale and Patil [34] developed an optimized vision transformer approach for classifying skin tumors with high accuracy. Their method involves preprocessing images to maintain color consistency and remove noise, followed by segmentation using a self-sparse watershed algorithm. Features were then extracted using a hybrid technique, and classification was done using an improved vision transformer. Their approach, tested on the ISIC 2019 database, achieved impressive results, with a precision of 96.65%.

Pacheco and Krohling [3] proposed a metadata processing block that utilizes metadata to enhance data classification and introduced an approach that combines both images and metadata using an attention-based mechanism within deep learning models to enhance the accuracy of skin cancer classification. The proposed model integrates CNN with attention layers that focus on important image features and relevant metadata, improving the ability of the models to differentiate between benign and malignant lesions. Experimental results demonstrated that the attention-based model significantly outperformed traditional image-only models in terms of classification accuracy, sensitivity, and specificity. Rezaee and Zadeh [35] proposed a bi-branch parallel model that integrates three key components: a Transformer module (TM), a self-attention unit (SAU), and a CNN. By combining the global feature extraction of the TM with the local feature extraction of the CNN through cross-fusion, the model generates fine-grained features for improved lesion identification. Additionally, an optimized lightweight optResNet-18 is introduced, achieving high accuracy in classifying skin cancer lesions, outperforming traditional CNNs and the TM on the ISIC-2019 and PH2 datasets with accuracies of 97.48% and 96.87%, respectively. The model also excels in state-of-the-art performance metrics, such as AUC, F1 score, specificity, precision, and recall.

Maurya *et al*. [36] introduced Dual AutoELM, an AI-based approach for identifying various skin cancers, and used two autoencoders: a spatial autoencoder for learning spatial features and an FFT-autoencoder for capturing textural and frequency patterns. Attention modules enhance feature learning, and an Extreme Learning Machine classifies skin malignancies based on features extracted from the autoencoders. Tested on the HAM10000 and ISIC-2017 datasets, the method showed high accuracy and robustness, with an AUC of 0.98 and accuracy of 97.66% for 'HAM10000' and an AUC of 0.95 and accuracy of 86.68% for ISIC-2017. Fan *et al*. [37] introduced the EAAC-Net, an efficient network for skin lesion segmentation. The network features two parallel encoders: the Efficient Adaptive Attention Module for global feature extraction with reduced computational complexity and the Efficient Multiscale Attention-based Convolution Module for enhancing local feature representation. Additionally, the Reverse Attention Feature Fusion Module is designed to progressively improve boundary information. EAAC-Net was tested on the ISIC 2016, ISIC 2018, and PH2 datasets, showing superior segmentation performance compared to other methods.

Recently, the Vision Mamba architecture introduced a novel bidirectional state space model that effectively captures visual information while minimizing the need for self-attention mechanisms, which are typically used in models like ViTs. This architecture enables Vision Mamba to deliver superior performance with greater computational efficiency, particularly in tasks, such as image classification, object detection, and semantic segmentation. Liu *et al*. [38] conducted a comprehensive survey that examined the application of Mamba across various visual tasks and data types, highlighting its evolution, recent advancements, and broad impact across multiple domains. Xu *et al*. [38] conducted an in-depth analysis of the Mamba architecture, delving into the details of representative visual Mamba backbone networks and their extensive applications. They also discussed the challenges and future directions, offering insights into new perspectives in this rapidly advancing field.

From the literature survey, it is understood that feature extraction, data augmentation, and model optimization techniques play significant roles in enhancing the performance of deep learning models for skin disease detection and classification. Still, challenges, such as class imbalance, data variability, and interpretability, remain areas of active research in skin disease diagnosis.

## 3 PROPOSED METHODOLOGY

In this section, we present the details of our work, including the acquisition of the dataset, preprocessing, finetuning of the models, and integration of the attention mechanism.

### 3.1 Acquisition of Dataset

Due to the availability of the benchmarking dataset, we downloaded the dataset of skin disease images from ISIC 2018^1^. In this dataset, the training input contains a total of 10,015 images of skin lesions with ground truth data for giving its types of skin lesions. The validation and testing datasets contain 193 and 1512 images, respectively. All the images have the corresponding ground truth data, which is used to classify the type of skin lesion images. This dataset has three skin cancers, Basal Cell Carcinoma (BCC), carcinoma/Bowen disease (AKIEC), and Melanoma (MEL), and four skin diseases, namely melanocytic nevi (NV), lichen-planus like keratosis (BKL), dermatofibroma (DF), and vascular lesions (VASC). This dataset not only provides visual representations of skin diseases but also includes the type of skin lesions for classifying multiclass labels, enhancing the potential for improving diagnostic accuracy. Fig. (**1**) shows a sample from every class.

^1^ https://challenge.isic-archive.com/data/#2018

### 3.2 Image Preprocessing and Augmentation

The primary goal of image processing is to improve the quality of the image and extract the information so that it can be interpreted more easily by machines or humans. İn our work, the following preprocessing techniques have been applied:

#### 3.2.1 Resizing

The images are resized to a fixed size suitable for the pre-trained models CNN. This ensures uniformity in the input dimensions.

#### 3.2.2 Normalization

The pixel values of images are normalized to a standard range, typically from o to 1, to help in faster convergence during training and ensure that the weights are updated efficiently.

#### 3.2.3 Image Augmentation

Since the number of images in each class is not balanced, after preprocessing the images, the dataset has been augmented by applying transformations, such as random rotations, flips, translations, and zooms, to generate additional training samples. This helps in improving the generalization ability of the models and reduces overfitting.

##### 3.2.3.1 Rotation

Images are rotated at random, up to a maximum of thirty degrees. By introducing diversity into the dataset, this rotation makes it possible for the models to learn from differences in the orientations of skin lesions.

##### 3.2.3.2 Horizontal Flip

Image flipping is done at random with a horizontal flipping tool. This augmentation increases the diversity of the training dataset by mirroring images along the horizontal axis.

##### 3.2.3.3 Vertical Flip

A random flipping of the vertical plane occurs. This augmentation adds variance by flipping images along the vertical axis, just like in horizontal flipping.

##### 3.2.3.4 Brightness Adjustment

Within a given range of [0.5, 1.5], random modifications are made to the brightness of images. With this adjustment, the model becomes more resilient to variations in image brightness.

The number of samples before and after augmentation of the dataset is presented in Table **1**. As there is a greater number of samples in the classes NV and BKL, no augmentation is applied over these two classes.

### 3.3 Attention Mechanism

Attention mechanisms enable models to selectively focus on specific parts of the input images, allowing for more effective feature extraction and improved performance on new datasets. The architecture of the pre-trained CNN models is modified to incorporate the attention mechanism, which typically involves adding attention layers to the models. Two mechanisms, namely spatial attention and channel-wise attention, have been used.

#### 3.3.1 Spatial Attention

This mechanism typically generates attention maps that indicate the importance or relevance of each spatial location in the input feature maps. These attention maps are then used to modulate the feature maps, emphasizing informative regions while suppressing irrelevant regions. Convolutional layers, followed by activation functions, are implemented to generate spatial attention weights for each spatial location. Then, the spatial attention module has been integrated into the CNN architecture by adding the attention module after specific convolutional layers in the network. By focusing on relevant spatial regions, spatial attention helps improve the localization and recognition capabilities of the models. Below, we present the steps involved in integrating spatial attention mechanisms.

Let X be the input feature produced by a convolution layer with dimension H*W*C representing height, width, and number of channels, respectively.

a. Compute attention weights α for each spatial location within the feature map by implementing a learnable function parameterized by weights Watt having convolutional and a fully connected layer.

α = Flearnable_fn (X, Watt)

b. Apply a softmax function along the spatial dimensions to ensure that the attention weights sum up to 1 across each channel:

α=softmax(α)

c. Compute the Attention-Weighted Feature Map by multiplying each element of the input feature map X by the corresponding attention weight α to obtain the attention-weighted feature map.

X_att_ (i,j,c)=X(i,j,c)×α(i,j,c)

d. Combine the attention-weighted feature map (X_att_) with the original feature map (X) to produce an enhanced representation that integrates spatial attention.

X_enhanced_ =concatenate(X,X_att_)

e. Pass the enhanced feature map *X*enhanced through an activation function Ø to introduce non-linearity and capture complex relationships between spatial features:

*Y*=Ø(*X*en_hanced_)

f. Pass Y through subsequent layers of the CNN to extract higher-level representations and make predictions.

#### 3.3.2 Channel-wise Attention

The channel-wise attention mechanism computes attention weights for each channel in the feature maps, indicating the importance of each channel in capturing task-specific information. These attention weights are then applied to the feature maps through element-wise multiplication, scaling the activations of each channel accordingly. To implement this attention mechanism, global pooling operations, followed by convolutional layers, are added to produce channel-wise attention weights. By attending to informative channels, channel-wise attention helps enhance the discriminative power of the models and improves their ability to capture fine-grained features. The steps are the same as spatial attention except step (iii), where we multiply each channel of the feature map X by the corresponding attention weight α to obtain the attention-weighted feature map.

To summarize, the spatial attention mechanism focuses on identifying relevant spatial regions within the skin images and finds the specific features or patterns in the images that are relevant for skin disease classification, such as lesion borders, texture variations, asymmetrical shapes, *etc*. On the other hand, the channel-wise attention mechanism focuses on identifying relevant channels or feature maps within the convolutional layers and accentuates important features or representations across different channels, thus enhancing the discriminative power of the models.

### 3.4 Model Training

This section outlines the procedures and methods employed in our work. Four pre-trained models, namely VGG16, ResNet152, EfficientNetB3, and RegNetX, have been utilized to develop the models for classification. These models have already been trained on the ImageNet dataset for image classification. During this pretraining phase, the models learned to extract useful features from images, gradually improving their ability to recognize different patterns and objects.

#### 3.4.1 Transfer Learning

After pretraining on a large dataset, these models are often used as starting points for other tasks, such as fine-tuning on a smaller dataset or for different tasks like object detection or image segmentation. This is known as transfer learning. Instead of training a model from scratch, which can be computationally expensive and requires a large amount of labeled data, transfer learning allows leveraging the knowledge learned by pretrained models on similar tasks. So, in our work, by leveraging the knowledge learned by the pretrained models, we developed a few models using RegNetX, EfficientNetB3, VGG19, and ResNet-152. This transfer learning enables faster convergence and better generalization on the skin dataset.

While performing transfer learning, the classification layers of the pretrained models are removed, leaving the earlier layers. These earlier layers serve as feature extractors, capturing abstract representations of the input data. The output of these layers is then used as the input features for the new models. In our work, we have fine-tuned a few top feature extraction layers. This helps the models to learn the features specific to the dataset under consideration. Then, classification layers are added on top of the feature extraction layers to create new models tailored to the skin dataset. These new layers typically include a few fully connected layers, followed by a softmax layer for the classification of 7 types of skin diseases. Table **2** shows the number of feature extraction layers finetuned and fully connected layers added to the classification block.

#### 3.4.2 Placement of Attention Modules

Spatial and channel-wise attention modules are typically integrated into the pre-trained models after convolutional layers. These layers are responsible for capturing spatial features, such as edges, textures, parts of the objects, *etc*. At this stage, the models have already learned some basic features but still have space to refine their understanding by focusing on specific regions of the images that are most useful for classification. In VGG19, the channel-wise attention modules are placed after a few convolutional blocks, especially after those responsible for higher-level feature extraction. In our case, these modules are placed after the eighth and ninth convolutional blocks. The spatial attention modules are placed before the max-pooling layer to improve the spatial focus before the feature maps are down-sampled. In ResNet-152, channel-wise attention modules are placed after each residual block to help the models emphasize the most informative channels, and spatial attention modules are placed before the addition operation in each residual block to refine spatial features that are being added to the residual connections. Furthermore, both channel-wise and spatial attention modules are placed before the final global average pooling layer to ensure that the models focus on the most relevant features across the entire feature map. Since RegNetX is divided into several stages, each containing a sequence of blocks, both attention modules are placed after each stage to enhance feature extraction at different levels of abstraction. Furthermore, these modules have also been placed after the bottleneck blocks to help refine the features both at a channel level and spatially. In the case of EfficientNetB3, channel-wise attention is integrated after each MBConv block to improve the focus on significant channels, and spatial attention modules are placed between MBConv blocks to enhance spatial feature selection before the next block processes the features. In addition, both modules are placed before the final pooling layer and classification head to ensure the models focus on the most important global features.

Furthermore, to tune the various hyperparameters, such as batch size, activation function, number of neurons, learning rate, and number of epochs, we used Bayesian optimization [39]. The search space for each hyperparameter, including its range or set of possible values, is defined, and the ideal values are chosen using Bayesian optimization. Fig. (**2**) shows the proposed workflow.

## 4 RESULTS AND DISCUSSION

During the experimentation phase, we performed a series of tests, initially without incorporating fine-tuning or attention modules, followed by experiments with fine-tuning and the inclusion of spatial and channel-wise attention. This approach was taken to assess the impact and importance of the attention module. For these experiments, we fine-tuned a few top feature extraction layers of the pre-trained models, such as VGG16, ResNet152, EfficientNetB3, and RegNetX, and Bayesian Optimization has been used for tuning hyperparameters. The results of the experiments are presented below:

### 4.1 Experimental Setup

The Keras model architectures were imported and initialized with pre-trained weights from ImageNet. Given the computational intensity and hardware demands of these models, we ran the models on Graphics Processing Units (GPUs). For VGG16, ResNet152, and RegNetX, all images were resized to dimensions 224x224x3, while for EfficientNetB3, resizing was done to 300x300. A total of 30 rounds of Bayesian optimization were conducted, each iteration comprising 100 epochs. The accuracy and loss metrics were tracked for each iteration. After analysis, the hyperparameters identified within 20 iterations were deemed optimal for our study, as further iterations did not yield significant improvement. Expanding the search space of the hyperparameters could potentially yield better hyperparameter values, but it would necessitate considerably more computational resources and time.

### 4.2 Experimental Results

We evaluated the performance of the finetuned VGG16, ResNet152, EfficietNetB3, and RegNetX using the optimized hyperparameters. Both attention mechanisms have been integrated into the models, and the results are presented below:

#### 4.2.1 Results of Spatial Attention Mechanism

First, we ran the finetuned models by integrating spatial attention, as discussed in Section 3. The results of such runs are presented in Tables **3** to **6**.

Next, we ran the developed models by integrating channel-wise attention into them, and the results are presented in Tables **7** to **10**.

Table **11** provides a summary of the performance of the fine-tuned models. It is evident from this table that RegNetX demonstrates superior performance compared to other models, achieving an accuracy of 98.61%.

We also used Cohen's Kappa coefficient, a statistical measure that assesses how much two classifiers agree while accounting for the possibility of chance agreement. This metric is commonly used to evaluate the performance of machine learning models in classification tasks. The Kappa coefficient ranges from -1 to 1, where -1 indicates complete disagreement, 0 indicates agreement by chance, and 1 represents perfect agreement. A higher Kappa value reflects better model performance. The Kappa score for the models we developed is mentioned in Table **12**.

As mentioned in Table **12**, all models showed high Kappa scores, indicating strong agreement between the classifiers and the actual labels, which suggests that the models performed well on the classification tasks. RegNetX has the highest Kappa scores (0.9637 for spatial attention and 0.9768 for channel-wise attention), indicating it has the best performance among the models in terms of agreement with the true labels. Furthermore, we also evaluated the performance of the fine-tuned models against existing models utilizing the ISIC dataset. The comparative results are mentioned in Table **13**. This table reveals that the models developed in this study generally perform at a level comparable to other existing models. Notably, among all the models examined, RegNetX stands out by outperforming not only the other models introduced in this research but also those that have been previously developed.

Using metrics, such as True Positive, True Negative, False Positive, and False Negative (new 1), we generated the confusion matrices, which are shown in Figs. (**3** and **4**).

### 4.3 Findings and Discussion

This study examined the potential of deep learning models in the detection and classification of skin diseases from dermoscopic images. By rigorously evaluating both traditional and modern deep learning architectures, including transfer learning techniques, we aimed to develop models that not only perform well but also generalize effectively to unseen data in automated skin disease diagnosis. While running the pre-trained models without integrating attention modules or finetuning, the results were found to be unappreciable.

#### 4.3.1 Key Findings

Our analysis revealed that the deep learning models excelled in accurately distinguishing between seven types of skin lesions, including melanoma, nevi, and benign lesions. Among the models we developed, RegNetX emerged as the top performer, achieving the highest accuracy. This success can be attributed to the modular design of RegNetX and regularity-based constraints, which enable it to excel where other models may struggle. Its architecture allows for high performance with fewer parameters, reducing the risk of overfitting and improving its generalizability to new, unseen data. We further enhanced the performance of RegNetX through fine-tuning, leveraging pre-trained knowledge to adapt to the specific characteristics of dermoscopic images. In addition, the optimization of hyperparameters, such as learning rate, batch size, and optimizer settings, significantly impacted the performance of deep learning models. Since the RegNetX has been fine-tuned more effectively with better-suited hyperparameters for the skin disease classification task, it leads to improved performance compared to other models.

To further refine feature extraction and increase detection accuracy, two attention mechanisms have been implemented. From the experiments, we understand that channel-wise attention performs comparatively better than spatial attention. As skin disease classification from dermoscopic images is a complex task that requires capturing subtle patterns and textures, the spatial attention mechanism failed to effectively identify relevant spatial regions within the images. Moreover, it struggled to discriminate between different skin lesions, leading to lower classification accuracy compared to the channel-wise attention mechanism. Meanwhile, channel-wise attention mechanisms are well-suited for capturing complex relationships among feature representations across channels, enabling the model to learn more abstract and discriminative representations of dermoscopic images. This enhanced capability to extract relevant features contributes to higher classification accuracy compared to spatial attention. Furthermore, the channel-wise attention mechanism facilitates better generalization across diverse skin lesion types and imaging conditions. By focusing on informative channels that capture common features shared among different types of lesions, channel-wise attention enables the model to learn robust representations that generalize well to unseen data. This improved generalization ability leads to higher accuracy in skin disease classification.

Since the Kappa score adjusts for the fact that some agreement between predictions and actual outcomes might occur by chance alone and makes it a more robust measure than simple accuracy, we have validated the models using this score, too, and the results are presented in Table **12**. From our experiments, we found that RegNetX with channel-wise attention produced the highest Kappa score compared to other models. RegNetX achieved the highest Kappa score due to its efficient modular design, effective fine-tuning, and the use of channel-wise attention mechanisms. Its architecture reduces overfitting and enhances generalization, leading to better feature learning and higher agreement with true labels. These factors combined make RegNetX the most effective model.

However, it is necessary to mention the limitations of our approach. While channel-wise attention showed superior performance, it may also lead to the loss of spatial information, especially in cases where fine-grained spatial details are important. For instance, lesions with intricate textures or shapes may not be captured effectively, which could impact the performance. Additionally, our reliance on dermoscopic images may limit the applicability of these models in real-world clinical settings where other imaging modalities or clinical data are used. Factors, such as image quality, dataset heterogeneity, and class imbalance, also demand further investigation to optimize performance.

#### 4.3.2 Ablation Study

In this section, we assess how different components or features of the models affect the overall performance of the classifiers. We achieved this by testing the classifiers without the integration of attention modules and fine-tuning. The results of these tests are mentioned in Table **14**. Since these models were trained on the ImageNet dataset, their performance on a new dataset was suboptimal. The pre-trained models might struggle to generalize to the new dataset if the types of features they learned from ImageNet do not match those relevant to the new dataset. However, after finetuning and integration of attention modules, the models performed well, as presented in Section 4.2.

## CONCLUSION AND SCOPE FOR FUTURE WORK

This study introduces a set of attention-aware deep learning models specifically designed for the classification of dermoscopic images aimed at improving the accuracy of skin disease diagnosis and classification. The models leverage the potential of attention mechanisms within deep learning architectures, which have been integrated into both traditional and contemporary CNN variants, including VGG16, ResNet152, EfficientNetB3, and RegNetX. Two types of attention mechanisms, spatial and channel-wise, were incorporated to enhance the ability of the models to focus on the most relevant features within the images. During the training process, Bayesian optimization was employed to identify optimal hyperparameter configurations, ensuring that each model was fine-tuned to achieve maximum performance. The experimental results demonstrated that the RegNetX model, when integrated with the channel-wise attention mechanism, consistently outperformed the other models developed in this study. Specifically, the improved classification accuracy with channel-wise attention is attributed to its enhanced capability to capture and leverage relevant feature representations across different channels, thereby improving the discriminative power of the model. This finding emphasizes the importance of channel-wise attention in effectively handling complex image data, suggesting that such mechanisms may be pivotal in advancing the state of the art in dermoscopic image classification.

To summarize, the integration of attention mechanisms into deep learning models represents a promising avenue for advancing the field of dermoscopic image classification and enhancing the accuracy of skin disease diagnosis. However, challenges, such as dataset bias, model interpretability, and real-world deployment, remain areas of ongoing research. Furthermore, the hybridization of spatial and channel-wise attention mechanisms would help to extract both spatial and element-wise features and can also effectively capture both local and global context information from the input images.

## Figures and Tables

**Fig. (1):**
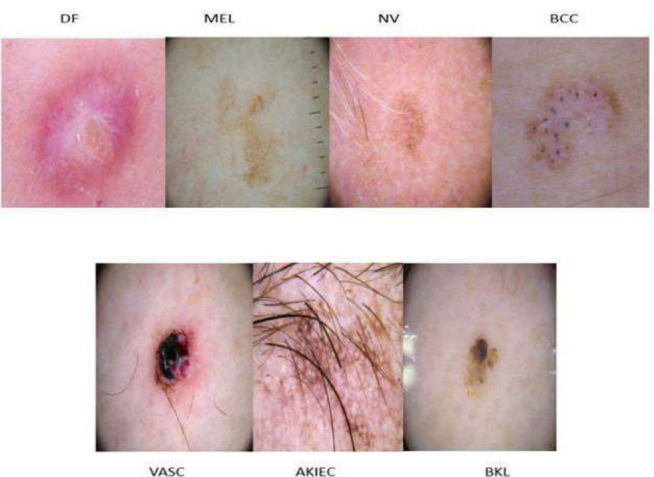
Sample images on the ISIC-2018 dataset.

**Fig. (2):**
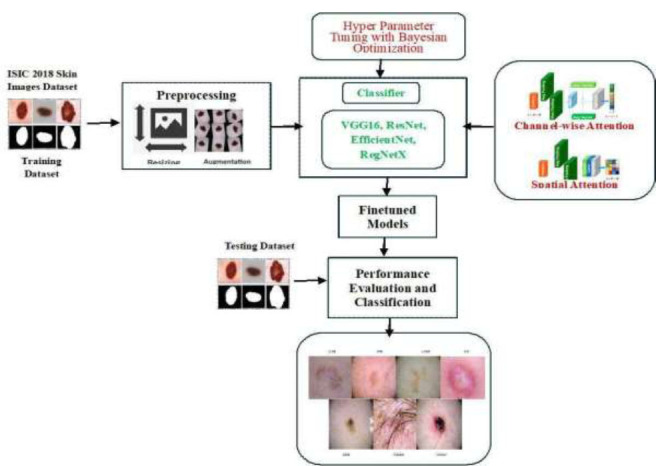
Proposed workflow.

**Fig. (3a-d):**
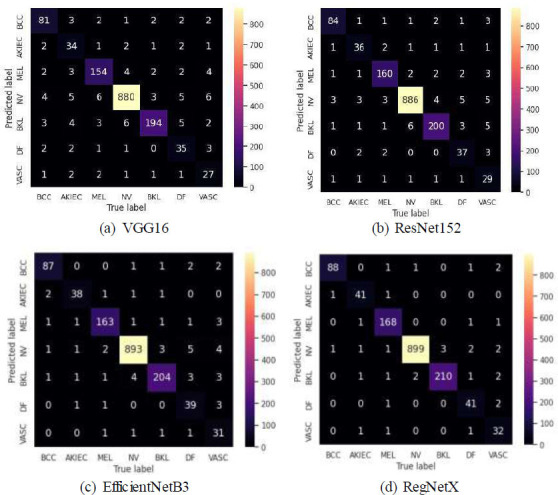
Confusion matrix for spatial attention.

**Fig. (4a-d):**
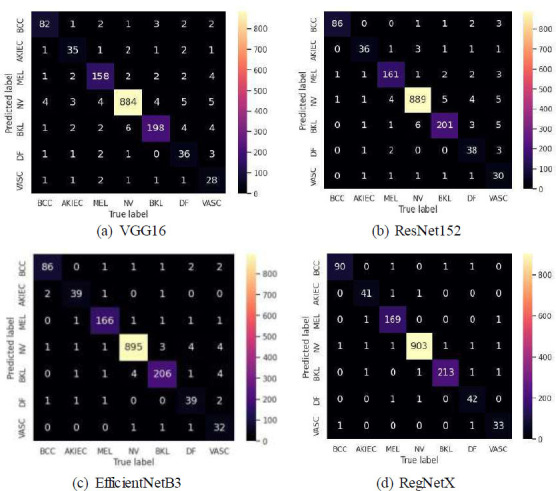
Confusion matrix for spatial attention.

**Table 1:** Class-wise samples of the dataset.

**Class**	**Number of Samples**		
	**Training**		**Testing**
	**Before Augmentation**	**After Augmentation**	
BCC	514	1020	93
AKIEC	327	1001	43
MEL	1113	1113	171
NV	6705	6705	909
BKL	1099	1099	217
DF	115	970	44
VASC	142	990	35

**Table 2:** Modification of pre-trained models.

**Pre-trained Models**	**Number of Layers Added**
VGG16	4 top layers and 2 fully connected + 1 Softmax
ResNet152	8 top layers and 2 fully connected + 1 Softmax
EfficientNetB3	8 top layers and 2 fully connected + 1 Softmax
RegNetX	8 top layers and 1 fully connected + 1 Softmax

**Table 3:** Performance of the models with spatial attention (VGG16).

**Class**	**Precision**	**Recall**	**F1 Score**
BCC	87.097	85.263	86.170
AKIEC	79.070	64.151	70.833
MEL	90.058	91.124	90.588
NV	96.810	98.324	97.561
BKL	89.401	95.567	92.381
DF	79.545	67.308	72.917
VASC	77.143	60.000	67.500
Accuracy	92.92		
Macro-F1	82.56		
Weighted-F1	92.69		

**Table 4:** Performance of the models with spatial attention (ResNet152).

**Class**	**Precision**	**Recall**	**F1 Score**
BCC	90.323	92.308	91.304
AKIEC	83.721	80.000	81.818
MEL	93.567	94.118	93.842
NV	97.470	98.774	98.117
BKL	92.166	95.238	93.677
DF	84.091	74.000	78.723
VASC	82.857	59.184	69.048
Accuracy	94.71		
Macro-F1	86.65		
Weighted-F1	94.54		

**Table 5:** Performance of the models with spatial attention (EfficientNetB3).

**Class**	**Precision**	**Recall**	**F1 Score**
BCC	93.548	94.565	94.054
AKIEC	88.372	90.476	89.412
MEL	95.322	96.450	95.882
NV	98.240	99.112	98.674
BKL	94.009	96.682	95.327
DF	88.636	76.471	82.105
VASC	88.571	67.391	76.543
Accuracy	96.23		
Macro-F1	90.29		
Weighted-F1	96.12		

**Table 6:** Performance of the models with spatial attention (RegNetX).

**Class**	**Precision**	**Recall**	**F1 Score**
BCC	94.624	97.778	96.175
AKIEC	95.349	91.111	93.182
MEL	98.246	96.552	97.391
NV	98.900	99.667	99.282
BKL	96.774	98.592	97.674
DF	93.182	87.234	90.110
VASC	91.429	78.049	84.211
Accuracy	97.82		
Macro-F1	94		
Weighted-F1	97.78		

**Table 7:** Performance of the models with channel-wise attention (VGG16).

**Class**	**Precision**	**Recall**	**F1 Score**
BCC	88.172	90.110	89.130
AKIEC	81.395	77.778	79.545
MEL	92.398	92.398	92.398
NV	97.250	98.551	97.896
BKL	91.244	94.737	92.958
DF	81.818	69.231	75.000
VASC	80.000	59.574	68.293
Accuracy	93.98		
Macro-F1	85.03		
Weighted-F1	93.81		

**Table 8:** Performance of the models with channel-wise attention (ResNet152).

**Class**	**Precision**	**Recall**	**F1 Score**
BCC	92.473	97.727	95.028
AKIEC	83.721	87.805	85.714
MEL	94.152	94.706	94.428
NV	97.800	98.668	98.232
BKL	92.627	95.261	93.925
DF	86.364	74.510	80.000
VASC	85.714	60.000	70.588
Accuracy	95.3		
Macro-F1	88.27		
Weighted-F1	95.15		

**Table 9:** Performance of the models with channel-wise attention (EfficientNetB3).

**Class**	**Precision**	**Recall**	**F1 Score**
BCC	92.473	95.556	93.989
AKIEC	90.698	90.698	90.698
MEL	97.076	97.076	97.076
NV	98.460	99.224	98.840
BKL	94.931	96.714	95.814
DF	88.636	81.250	84.783
VASC	92.473	95.556	93.989
Accuracy	96.76		
Macro-F1	91.6		
Weighted-F1	96.69		

**Table 10:** Performance of the models with channel-wise attention (RegNetX).

**Class**	**Precision**	**Recall**	**F1 Score**
BCC	96.774	97.826	97.297
AKIEC	95.349	93.182	94.253
MEL	98.830	97.688	98.256
NV	99.340	99.669	99.504
BKL	98.157	99.070	98.611
DF	95.455	91.304	93.333
VASC	96.774	97.826	97.297
Accuracy	98.61		
Macro-F1	96.32		
Weighted-F1	98.6		

**Table 11:** Overall accuracy for spatial and channel-wise attention.

**Classes**	**Attention Mechanism**			
	**Spatial**		**Channel-wise**	
	**Validation Accuracy (%)**	**Testing Accuracy (%)**	**Validation Accuracy (%)**	**Testing Accuracy (%)**
**VGG16**	93.12	92.92	93.94	93.81
**ResNet152**	95.63	94.54	96.82	95.15
**EfficientsNetB3**	97.01	96.12	97.54	96.69
**RegNetX**	98.84	97.78	98.94	98.61

**Table 12:** Cohen's kappa coefficient for the developed models.

**Models**	**Kappa Score**	
	**Spatial**	**Channel-wise**
VGG16	0.8831	0.9004
ResNet152	0.9124	0.9211
EfficientsNetB3	0.9374	0.9461
RegNetX	0.9637	0.9768

**Table 13:** Performance comparison of the developed models.

**Models**		**Accuracy (%)**
**Proposed Models/Ref.**	**VGG16**	93.81
	**ResNet152**	95.15
	**EfficientsNetB3**	96.69
	**RegNetX**	**98.61**
Cai *et al*. [2]	ViT model	93.81
Antari *et al*. [31]	DenseNet-201 Inception-V3 InceptionResNet-V2ResNet-50	84.6084.7085.8090.10
Milton [32]	PNASNet-5-Large InceptionResNetV2	76.070.0
	SENet154InceptionV4	74.067.0
Naeem *et al*. [40]	SNC_net	97.81
Sadik *et al*. [41]	Xception	97.0
Ghosh *et al*. [42]	DenseNet121	91.82
Ahammed *et al*. [24]	SVM, KNN, DT	95
Rashid *et al*. [43]	MobileNetV2	98.2
Choudhary *et al*. [44]	DNN	84.45
Gouda *et al*. [45]	Customized CNN Resnet50InceptionV3 Inception Resnet	83.283.785.884

**Table 14:** Results of the ablation study.

**Classes**	**Accuracy**
VGG16	82.62
ResNet152	83.29
EfficientsNetB3	85.18
RegNetX	88.53

## Data Availability

The data from the manuscript is available on reasonable request to the corresponding author [J.C].

## LIST OF ABBREVIATIONS

AI: Artificial Intelligence
CNNs: Convolutional Neural Networks
KNN: K-Nearest Neighbors
GLCM: Gray Level Cooccurrence Matrix
TM: Transformer module
MEL: Melanoma
BSC: Basal Cell Carcinoma
DF: dermatofibroma
